# Unveiling a novel clinical risk assessment model for identifying non-suicidal self-injury risks in depressed Chinese adolescents amidst the COVID-19 pandemic: insights from low self-esteem, internet use, and sleep disturbance

**DOI:** 10.3389/fpsyt.2023.1259909

**Published:** 2024-01-05

**Authors:** Zhongyi Liu, Yuhuan Wei, Ying Yang, Linghua Kong

**Affiliations:** ^1^Department of Psychiatry, School of Clinical Medicine, Cheeloo College of Medicine, Shandong University, Jinan, China; ^2^Childhood Psychiatry Unit, Shandong Mental Health Center, Jinan, China; ^3^School of Mental Health, Jining Medical University, Jining, China; ^4^School of Nursing and Rehabilitation, Cheeloo College of Medicine, Shandong University, Jinan, China

**Keywords:** depressive disorder, sleep disturbance, self-esteem, non-suicidal self-injury, adolescent

## Abstract

**Background:**

Non-suicidal self-injury (NSSI) is a highly prevalent behavioral problem among depression adolescent patients that can result in numerous adverse outcomes. This study endeavors to bridge this knowledge gap by creating a comprehensive model that incorporates multiple aspects of NSSI to accurately evaluate its risk in adolescents with depression, thereby enhancing our ability to prevent and address this challenging issue.

**Method:**

Using a cross-sectional design, we recruited 302 adolescents with depressive disorders who visited or were hospitalized at Shandong Mental Health Center from December 2021 to June 2022. The participants completed several self-report questionnaires, including the Chinese version of the Internet Addiction Test, the Pittsburgh Sleep Quality Index questionnaire, the Defeat Scale, the Social Avoidance and Distress Scale and the Children’s Depression Inventory. Logistic regression analysis was performed to identify the diagnostic factors, which were further used to establish clinical risk assessment models. A receiver operating characteristic curve (ROC) to identify the best model. An external validating team was introduced to verify the assessing efficiency.

**Results:**

Based on a logistic regression analysis, three variables have been identified as significant risk factors. Specifically, adolescents with depression who experience low self-esteem, internet use, or suffer from sleep disturbance face an increased risk of NSSI. An integrated risk index for NSSI exhibits excellent accuracy in identifying depressed adolescents at risk of NSSI (area under the curve = 0.86, sensitivity = 0.88, specificity = 0.69). In the validation cohort, the identification performance remains strong (area under the curve = 0.84, sensitivity = 0.72, specificity = 0.81).

**Conclusion:**

This study highlighted the role of self-esteem, internet use and sleep disturbance in the development of NSSI. The risk index diagnosing NSSI onset may help to guide the design and application of novel interventions to minimize this risky behavior in future depressed adolescents.

## 1 Introduction

Non-suicidal self-injury (NSSI) is the direct, deliberate destruction of one’s body organs or tissues (e.g., cutting, burning) in a socially unapproved manner, without suicidal intent (1). The Diagnostic and Statistical Manual of Mental Disorders (DSM-5) criteria for NSSI include engaging in such behaviors for 5 days or more in the past year, along with evidence indicating associated distress, impairment, or disruption to daily functioning (2). Nearly Chinese adolescents, around 29% of adolescents had experienced NSSI within a year (3), and the prevalence of NSSI among depressed adolescents in China is as high as 62.2% (4). Although NSSI is a behavior without suicidal intentions, it was associated with the risk of subsequent suicide behavior. The risk of suicide in the NSSI group is seven times higher in adolescents with depression than in adolescents without NSSI (4–7). In many cases, clinicians are unable to recognize the onset of NSSI in time because patients deliberately conceal such actions and may not leave visible marks on the body. This highlights the challenge of accurately detecting NSSI and the need for improved assessment strategies in clinical practice. An effective tool is required to detect early signs of NSSI in depressed adolescents for timely intervention.

In our clinical observations, we categorized factors influencing NSSI into behavior disorders, biological factors, and clinical traits. Notably, the interplay of these factors becomes more intricate when influenced by large-scale social events such as the COVID-19 pandemic. The Pandemic’s containment measures have led to reduced social interaction among adolescents with depressive disorders, exacerbating maladaptive coping mechanisms (8) and potentially intensifying psychological stress (9) and impeding adolescent development (10). During the COVID-19 pandemic, adolescents with depression showed reluctance to interact with others and go to school, while preferred to spend time online (11). Several prior studies have demonstrated the relationship between sleep deprivation and prolonged use of the internet (12, 13). Additionally, findings from a study conducted in China suggest that internet use indirectly influences suicidal ideation through its impact on sleep patterns (14), thereby causing serious consequences. Lifestyle changes also lead to lower academic performance and often lead to increased academic frustration. Previous studies have indicated that adolescents engaging in NSSI during the COVID-19 outbreak exhibit elevated levels of depression, increased dissatisfaction with their academic performance, and a heightened sense of frustration. Additionally, the NSSI group demonstrates lower self-esteem compared to their non-NSSI counterparts, and they also exhibit diminished levels of social support and interpersonal engagement (15). According to the available literature, it can be inferred that various factors, including frustration, social avoidance, and internet addiction, are associated with an increased risk of NSSI behavior (15–17). Some of the above unpleasant emotional experiences bring painful feelings to patients. In order to ‘redeem’ themselves, they choose NSSI to relieve their pain and to make themselves feel better. The Experiential Avoidance Mode (EAM) suggested that NSSI is primarily maintained by negative reinforcement in the form of avoidance or avoidance of unwanted emotional experiences (18). Apparently, these factors that elicit distressing emotional experiences can serve as early indicators of NSSI in depressed adolescents.

Previous investigations assessing the initial pathogenesis of NSSI have been hampered by several limitations. Prior research has commonly focused on populations unaffected by the COVID-19 pandemic or prolonged periods of quarantine. Some studies have only focused on single-sex samples when exploring behavioral diagnostic factors for NSSI. Additionally, the small sample sizes used in studies involving adolescents with depressive disorders may limit the generalizability of the findings. Furthermore, many investigations have had a narrow coverage of risk factors, failing to encompass the multiple facets that contribute to NSSI onset. The present study aims to overcome these limitations by conducting a cross-sectional examination of multiple potential risk factors associated with the development of NSSI among adolescents with depressive disorders. The study examined behavior disorder, biological factors, as well as three clinical traits, namely frustration, depression, and social avoidance, in order to evaluate their respective contributions to the risk of NSSI onset.

Considering the emergence of behavior disorders, biological factors, and three clinical traits (namely frustration, depression, and social avoidance) as potential risk factors for NSSI, our hypothesis was that by incorporating the aforementioned factors, we could formulate a clinical model capable of effectively evaluating the presence of NSSI behaviors in depressed adolescents. To this end, the present study aimed to devise a novel clinical model that could serve as a decision-making tool to assess NSSI behaviors in this specific population.

## 2 Methods

### 2.1 Participants and procedures

This study was a cross-sectional study, and to achieve clinical effectiveness validation, two sub samples of adolescent depression patients were collected at different time periods using sequential enrollment, defined as the test set and validation set. Test set: a total of 302 adolescent depressive disorder patients, 74 males and 228 females; age 10–18 years, mean 15.1 (SD = 1.8) years, attending outpatient clinics and hospitalization at Shandong Mental Health Center from December 2021 to June 2022 were selected using convenience sampling. Diagnostic talks and assessments of the enrolled patients were conducted by two psychiatrists specializing in psychiatry with the title of attending or above using the Childhood Affective Disorders and Schizophrenia Definitive Examination Questionnaire Lifetime (K-SADS-PL), and all study personnel involved in the assessment were trained in consistency. Inclusion criteria: (1) meeting the DSM-5 diagnostic criteria for depressive disorders; (2) age 10–18 years; (3) being able to cooperate in completing the questionnaire and scale evaluation related to this study; and (4) obtaining informed consent from the patients and their legal guardians. Exclusion criteria: (1) co-morbidities with other psychiatric disorders, such as neurodevelopmental disorders (autism spectrum disorder, attention deficit hyperactivity disorder, etc.); (2) co-morbidities with severe physical illness; (3) history of suicide attempts (e.g., jumping from a building, hanging oneself, swallowing high doses of drugs, etc.) in the past 1 month. NSSI was assessed according to the DSM-5, and patients were defined as having NSSI if one of the following two criteria was met: (1) patients had ≥3 self-harm in the past 6 months, including at least 1 in the last 1 month; (2) patients had ≥5 self-harm in the past 1 year, including at least 1 in the last 1 month. One hundred and seventy-eight cases in the group with NSSI and 124 cases in the group without NSSI were obtained.

Validation Sets using the same inclusion and exclusion criteria as in the experimental set, 164 patients were included in outpatient visits and hospitalizations from June to September 2022 in Shandong Province Mental Health Center. Among the included patients, 59 were male and 105 were female. The mean age was 15.1 (SD = 1.8) years, and 87 cases in the group with NSSI and 77 cases in the group without NSSI were obtained.

### 2.2 Measurements

#### 2.2.1 Demographic variables

A self-designed questionnaire was used for this study, including gender (male/female), age (10–18 years old), grade of schooling (junior high school and below/high school and above), family history of psychosis (with/without), family location (urban/rural), have siblings or not (yes/no), marital status of biological parents (normal/divorced/unilateral alive), and financial status (well/medium/poor).

#### 2.2.2 Children’s depression inventory

The scale was developed by Kovacs (19) to assess depressed mood or behavior in adolescents in the past 2 weeks. There are 27 items with 5 subscales: negative mood, interpersonal problems, ineffectiveness, lack anhedonia and low self-esteem. The scale is rated on a 3-point scale from 0 to 2, with higher scores indicating greater depression. The scale has been shown to have good reliability indicators with a Cronbach’s alpha coefficient of 0.93.

#### 2.2.3 The Pittsburgh sleep quality index questionnaire

The sleep disturbance dimension of The Pittsburgh Sleep Quality Index questionnaire developed by Buysse et al. (20) was used to measure the degree of sleep disturbance in individuals’ nighttime sleep. The scale’s Chinese version was revised by Liu et al. (21). The sleep disturbance dimension consists of nine items designed to assess the degree of sleep disturbance in subjects, including nine topics such as the presence of early awakening, the presence of getting up to go to the toilet, uncomfortable breathing, loud coughing or snoring, and feeling cold. The dimension is scored on a 4-point scale from 0 (none) to 3 (3 times/week), with higher scores indicating a more severe level of trauma experienced. The Chinese version of the scale has a Cronbach’s alpha coefficient of 0.82.

#### 2.2.4 The internet addiction test

Internet use was measured using the Chinese version of the Internet Addiction Test (IAT), which is widely used when assessing internet addiction (22). This has been widely used to measure various types of Internet addiction (23). The use of this scale is designed to measure the extent to which an individual is dependent on internet use. This scale a six-point scale (0 = Never; 1 = Seldom; 2 = Occasionally; 3 = Frequently; 4 = very often; 5 = always). Higher scores indicate greater reliance on the network. The Cronbach’s alpha in this study was 0.93.

#### 2.2.5 Defeat scale

This study measured the level of defeat of patients using Defeat Scale developed by Gilbert and Allen (24). This scale a five-point scale (0 = Never; 1 = Seldom; 2 = Occasionally; 3 = Frequently; 4 = always). This scale is used to measure a person’s level of frustration. Some of the options use reverse scoring. With higher scores representing higher levels of defeat for the test taker. The Cronbach’s alpha in this study was 0.96.

#### 2.2.6 Social avoidance and distress scale

This study measured patients’ social avoidance using the Social Avoidance and Distress Scale developed by Watson and Friend (25). The scale’s Chinese version was revised by Chunzi et al. (26). The scale is answered yes or no, and scores are recorded according to the way the answer corresponds to each question. The scale is also divided into a social avoidance subscale and a social distress subscale. The higher the total score, the higher the corresponding level of social avoidance and distress. The Cronbach’s alpha in this study was 0.94.

### 2.3 Data analyses

SPSS statistics 26.0 and R Studio 4.2.0 statistical software for general demographic information and clinical data, with statistical significance set at *p* < 0.05. The main statistical methods adopted in this study are outlined below:

#### 2.3.1 Descriptive analysis

General demographic information of the study subjects was described, and the incidence of Non-Suicidal Self-Injury (NSSI) behavior in adolescents with depressive disorders was analyzed. Normality tests were conducted for continuous data, and those conforming to a normal distribution were expressed as mean ± standard deviation (x ± s), while categorical data were presented as frequencies and percentages.

#### 2.3.2 Chi-square test

Comparative analyses were conducted on groups with and without NSSI concerning gender, education level, family history, place of residence, only child status, parents’ marital status, and family economic status using the chi-square test.

#### 2.3.3 Independent samples *t*-test

Differences in depression levels, internet use, social avoidance, frustration, and sleep disturbance scale scores were analyzed between groups with and without NSSI using independent samples *t*-tests.

#### 2.3.4 Binary logistic regression

In the test group, binary logistic regression analysis was employed to explore the independent risk factors for NSSI behavior after adjusting for general information such as gender, age, education level, family history, place of residence, only child status, parents’ marital status, and family economic situation. The focus was on understanding the independent impact of depression levels, internet use, social avoidance, frustration, and sleep disorders on NSSI behavior.

#### 2.3.5 Construction of a risk assessment model

Using the Receiver Operator Characteristic curve, the joint predictive ability of factors with significant independent contributions in logistic regression was evaluated for the risk assessment of NSSI in adolescents with depressive disorders.

## 3 Results

### 3.1 Participants’ characteristics

A total of 302 depressed adolescents in the test set met the inclusion criteria, of which 178 (58.9%) had NSSI behavior (with NSSI group) and 124 (41.1%) had no NSSI behavior (without NSSI group). There were no statistically significant differences in gender, age, education, family location, sibling, marital status of biological and financial status in the test set; there were statistically significant differences in family history (Table 1).

**Table 1:** Descriptive characteristics of the study participants (*n* = 302).

Variable	With NSSI *n* = 178 (%)	Without NSSI *n* = 124 (%)	*χ*^2^/*t*
Gender			2.33
Male	38 (29.0)	36 (21.3)	
Female	140 (71.0)	88 (78.7)	
Education			0.82
Junior high school and below	81 (45.5)	63 (50.8)	
High school and above	97 (54.5)	61 (49.2)	
Family history of psychosis			8.87^*^
Without	152 (85.4)	115 (96.0)	
With	26 (14.6)	5 (4.0)	
Family location			0.14
Urban	120 (67.4)	81 (65.3)	
Rural	58 (32.6)	43 (34.7)	
Have siblings or not			0.91
YES	58 (32.6)	47 (37.9)	
NO	120 (67.4)	77 (62.1)	
Marital status of biological			3.30
Normal	152 (85.4)	113 (91.1)	
Divorced	21 (11.8)	7 (5.6)	
Unilateral alive	5 (2.8)	4 (3.2)	
Financial status			1.06
Well	32 (18.0)	28 (22.6)	
Medium	109 (61.2)	70 (56.5)	
Worse	37 (20.8)	26 (21.0)	
	Mean ± SD	Mean ± SD	
Age	15.0 ± 1.7	15.1 ± 1.8	−0.41

NSSI, patients with non-suicidal self-injury. **p < 0.001. *p < 0.05. M ± SD: Mean ± Standard Deviation. Group comparisons: *t*-test or chi-square test.

### 3.2 Comparison of scores on various scales in two groups of adolescents with depression

Comparison of the two groups of patients in the test set showed statistically significant differences in the CDI, DS, SAD and IAT scores for each factor, and in the total CDI, DS, SAD, IAT and sleep disturbance scores (all *p* < 0.01), and all scores were higher in the group with NSSI than in the group without NSSI (Table 2).

**Table 2:** Differences in SAD, CDI, IAT, sleep disturbance and DS between the depressed group without NSSI and the depressed group with NSSI.

The scale	With NSSI *n* = 178	Without NSSI *n* = 124	*t*	*p*
Social distress	11.3 ± 3.3	7.9 ± 4.5	−7.05	<0.001
Social avoidance	10.8 ± 3.5	7.6 ± 4.1	−7.01	<0.001
CDI 1	6.8 ± 3.3	3.5 ± 3.3	−8.71	<0.001
CDI 2	2.9 ± 1.8	1.6 ± 1.3	−7.02	<0.001
CDI 3	6.2 ± 2.1	3.3 ± 2.1	−12.01	<0.001
CDI 4	5.5 ± 1.7	3.9 ± 2.1	−7.08	<0.001
CDI 5	8.9 ± 3.5	5.5 ± 3.5	−8.38	<0.001
IAT	51.5 ± 16.7	38.9 ± 15.7	−6.64	<0.001
Sleep disturbance	5.5 ± 1.4	5.9 ± 2.1	−9.03	<0.001
DS	42.4 ± 13.9	27.6 ± 18.0	−5.30	<0.001

*N* = 302, NSSI, patients with non-suicidal self-injury. CDI: Children’s Depression Inventory, IAT: The Internet Addiction Test, DS: Defeat Scale, CDI 1 = CDI negative mood, CDI 2 = CDI interpersonal problems, CDI 3 = CDI low self-esteem, CDI 4 = CDI ineffectiveness, CDI 5 = CDI anhedonia. Group comparisons: *t*-test.

### 3.3 Logistic regression analysis of factors influencing NSSI behavior in depressed adolescents

Using with and without NSSI as dependent variables and factors that differed in univariate analysis as independent variables, binary logistic stratified regression analysis showed that family history was significantly associated with NSSI in adolescents with depression in general clinical data (OR = 4.75, *p* = 0.02, 95% CI: 1.34 to 16.87), grade of schooling was significantly associated with adolescents with depression NSSI (OR = 3.46, *p* = 0.02, 95% CI: 1.24–9.71), low self-esteem in CDI (OR = 2.05, *p* < 0.001, 95% CI: 1.58–2.65), and sleep disturbance (OR = 1.07, *p* = 0.02, 95% CI: 1.00–1.147), IAT (OR = 1.03, *p* < 0.01, 95% CI: 1.01–1.05) contributed independently to the emergence of NSSI behaviors in adolescents with depression in a statistically significant manner (Table 3).

**Table 3:** Stratified regression of NSSI behaviors in adolescents with depressive disorders.

	Model 1	Model 2	Model 3
Variable	OR (95% CI)	OR (95% CI)	OR (95% CI)
**Gender**			
Male	1	1	1
Female	1.60 (0.93–2.75)	1.53 (0.88–2.66)	1.07 (0.51–2.25)
Age	0.83 (0.67–1.02)	0.82 (0.66–1.02)	0.94 (0.70–1.26)
**Education**			
Junior high school and below	1	1	1
High school and above	2.31 (1.10–4.85)*	8.89 (2.58–30.63)*	3.46 (1.24–9.71)*
**Family history of psychosis**			
Without	1	1	1
With	4.32 (1.60–11.72)*	3.95 (1.432–10.88)*	4.75 (1.34–16.87)*
**Family location**			
Urban	–	1	1
Rural	–	0.95 (0.56–1.64)	0.81 (0.39–1.66)
**Have siblings or not**			
YES	–	1	1
NO	–	0.73 (0.42–1.25)	0.80 (0.38–1.65)
**Marital status of biological**			
Normal	–	1	1
Divorced	–	2.15 (0.83–5.57)	2.11 (0.63–6.99)
Unilateral alive	–	0.87 (0.21–3.61)	1.14 (0.17–7.44)
**Financial status**			
Well	–	1	1
Medium	–	1.28 (0.70–2.36)	1.14 (0.17–7.44)
Worse	–	1.18 (0.56–2.51)	1.14 (0.17–7.44)
Social distress	–	–	1.05 (0.93–1.19)
Social avoidance	–	–	1.01 (0.89–1.15)
CDI 1	–	–	1.01 (0.88–1.17)
CDI 2	–	–	1.08 (0.84–1.40)
CDI 3	–	–	2.05 (1.58–2.66)**
CDI 4	–	–	0.99 (0.78–1.25)
CDI 5	–	–	0.96 (0.82–1.19)
IAT	–	–	–
Sleep disturbance	–	–	–
DS	–	–	0.97 (0.93–1.00)
Constants	10.60	11.060	0.023
Model *χ*^2^ (*p*)	17.17*	4.328	133.48**
Block *χ*^2^ (*p*)	17.17*	21.501*	154.98**
Nagelkerke *R*^2^	0.08	0.09	0.54

NSSI, patients with non-suicidal self-injury. CDI: Children’s Depression Inventory, IAT: The Internet Addiction Test, DS: Defeat Scale. CDI 1 = CDI negative mood, CDI 2 = CDI interpersonal problems, CDI 3 = CDI low self-esteem, CDI 4 = CDI ineffectiveness, CDI 5 = CDI anhedonia. **p < 0.001. *p < 0.05. Model 1: Gender, Age, Education, Family history of psychosis. Model 2: Model 1+ Family location, Have siblings or not, Marital status of biological, Financial status. Model 3: Model 2+ Social distress, Social avoidance, CDI negative mood, CDI interpersonal problems, CDI low self-esteem, CDI ineffectiveness, CDI anhedonia. The Internet Addiction Test, Sleep disturbance, Defeat Scale.

### 3.4 Assessment and analysis of the impact of risk factors on NSSI behavior

**Figure 1:**
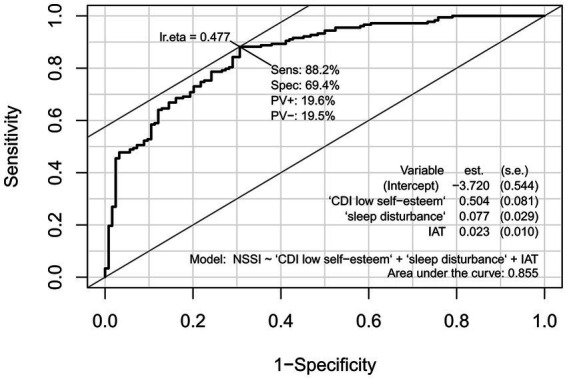
ROC curve for the risk factors for NSSI in Testing Group.

**Figure 2:**
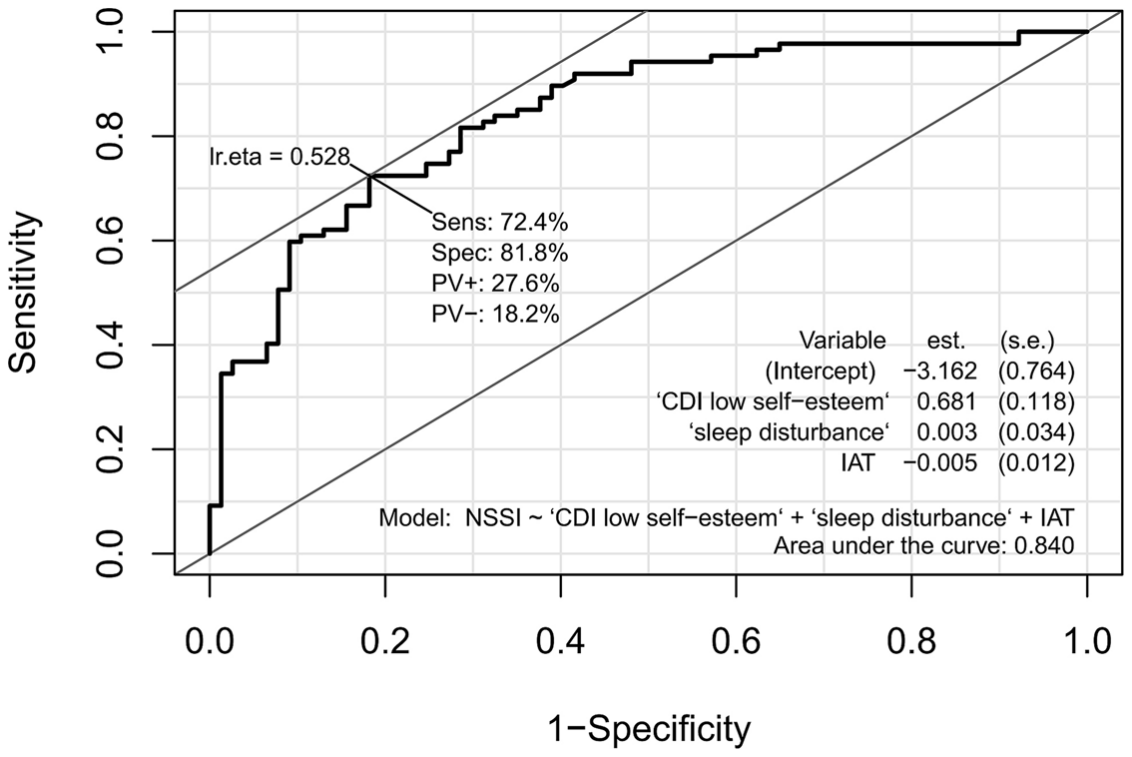
ROC curve for the risk factors for NSSI in Validation Group.

The above model was analyzed for diagnostic value using ROC curves based on the CDI low self-esteem factor scores, IAT scores and sleep disturbance scores for both groups of subjects (Figure 1). The results showed that the AUC = 0.86, 95% CI: 0.81 to 0.90, *p* < 0.001 and Yoden’s index = 0.48. To verify the validity of the clinical diagnostic model, the above model was applied to the new test set and the corresponding ROC curve was obtained by calculating the true probability values (Figure 2), AUC = 0.84, 95% CI: 0.78 to 0.90, *p* < 0.001, Yoden’s index = 0.53. These results indicated that the CDI low self-esteem factor scores, IAT scores and sleep disturbance scores had a high evaluate efficiency for the presence in NSSI behavior in adolescents with depressive disorders.

## 4 Discussion

The present study developed a clinical risk assessment model for NSSI among depressed adolescents during the COVID-19 pandemic. This study found that NSSI could be accurately identified by family history of psychosis, behavior disorder, biological factors, and clinical traits, which include low self-esteem, sleep disorder and internet use. Specifically, sleep disturbances, low self-esteem, and internet use emerged as significant diagnostic factors for NSSI in depressed adolescents. These factors effectively contribute to evaluating the likelihood of NSSI behaviors in this population.

First and foremost, our study revealed a significant association between low self-esteem scores and the incidence of NSSI among adolescents with depression, which is consistent with previous findings (27, 28). Low self-esteem is a clinical manifestation of depression, and higher levels of low self-esteem tend to mean higher levels of depression (29). As a risk factor for NSSI, depressive disorders also play a role in the development of NSSI (30, 31). Adolescents with depression are more likely to encounter challenging situations at home or in an educational setting that they cannot resolve and get stuck in. These issues may include childhood trauma, school violence, and academic stress, among others (32–34), and it is possible to exacerbate and interact with low self-esteem. Adolescents with depression and low self-esteem may harbor feelings of hopelessness and helplessness, compelling them to resort to NSSI as a means to gaining attention from others. In some cases, these individuals may engage in NSSI to demonstrate their superiority over others and to earn admiration and respect, while also seeking out social connections through increased interaction with peers. During the pandemic of COVID-19, it is also important to consider the impact of weight gain during lockdown as a contributing factor (35). With the closure of parks, gyms, and hiking trails, individuals are experiencing more sedentary lifestyles, increased snacking, and overeating (36). These lifestyle changes have led to weight gain, which can cause patients to be more self-conscious than usual. And it does not just happen in adolescents. It is predictable that these patients will continue to have a higher probability of developing NSSI behavior if they do not improve their low self-esteem symptoms in a timely and effective manner. This underscores the importance of addressing and managing low self-esteem symptoms in adolescents with depressive disorders.

At night, children burdened with low self-esteem are often restless, tossing and turning, and having difficulty falling asleep while suffering in silence. This emotional torment not only affects their mental well-being, but frequently manifests itself in physical discomfort. This study sheds light on the crucial role of sleep disturbance as a significant factor contributing to the development of NSSI among adolescents with depressive disorders. The adverse impact of sleep disorders on mental health and their potential to give rise to physical and psychological issues were well recognized (37–39). Healthy sleep plays a vital role in the development of young people (40), and previous studies have indicated a significant correlation among frequent nightmares, insomnia, sleep deprivation, and NSSI behavior in adolescents (41, 42). The implementation of COVID-19 quarantine measures has led to weight gain (35), which could potentially contribute to obstructive sleep apnea and be an underestimated factor in sleep disorders. The existence of a sleep disorder can disrupt an individual’s regular sleep pattern, which can affect the function of various regions in the adolescent brain. Of note, the orbitofrontal cortex and dorsolateral prefrontal cortex are thought to be particularly vulnerable to changes in sleep patterns. The orbitofrontal cortex is known to be involved in depression and other psychiatric disorders, and changes in this region may contribute to the development of NSSI behaviors in addition to affecting depression. The orbitofrontal cortex is known for its involvement in depression and other psychiatric disorders, and changes in this area can contribute to the onset of NSSI behaviors in addition to affecting depression (43). Depressed teens with poor sleep quality may have difficulty concentrating during the day, leading to lower academic performance. This may further fuel parental resentment and increase discrimination by peers and teachers in the academic setting, ultimately leading to low self-esteem and emotional distress in affected individuals. For pain relief, those suffering may turn to NSSI for comfort, redemption, and eventual release from their inner torment.

Insomnia and sleep disorders are common among teenage patients with depressive disorders, and it is often during sleepless nights that they turn to the internet for solace and distraction (12). In this study, research shows an increase in Internet use during the COVID-19 pandemic, including the frequency and duration of recreational Internet use and the frequency of late-night Internet use (44, 45). The information cocoon effect of the internet big data has important implications for the mental health of young people with depressive disorders. Previous study had shown that such individuals are more likely to browse the internet for information related to depression and self-harm, seeking empathy and connection with others who share their struggles (46). However, increased time spent online can also lead to conflicts between family members and patients, as patients become more entrenched in the digital world and disconnected from real-life interactions. Some patients may experience a distorted sense of reality, characterized by a feeling of unreality and haziness. To ground themselves in reality, these individuals may engage in NSSI. Moreover, some patients who have become addicted to the online world may experience greater distress when confronted with real-life challenges, leading them to turn to NSSI as a means of coping with their suffering. Additionally, the use of the internet may exacerbate feelings of resentment and thoughts of revenge against family members, further contributing to NSSI behavior among those struggling with depressive disorders. A large-scale survey conducted on Internet addiction and NSSI behavior among Chinese adolescents revealed a correlation between NSSI and Internet addictive behavior (17).

However, some of the results of this study contradict the findings of other studies. Some studies showed that NSSI was associated with social avoidance and frustration (47–49), whereas the present study showed that social avoidance and frustration was not associated with NSSI. It is considered to be related to the experience of being in experiencing home quarantine. The inconsistencies in the results of previous studies may be related to different characteristics of study populations, study designs and adjustments, and different measurements and standards.

The limitations of this study must be acknowledged. First, the sample size was relatively small, and the study was conducted in a single hospital, which may limit the generalizability of the findings to other populations. Second, the study was cross-sectional, which means that it is difficult to establish a causal relationship between the variables. Third, further longitudinal studies are needed to investigate the dynamic relationships between depressive disorders, sleep disturbance, internet use, and NSSI behavior among adolescents. It is also important to consider the potential impact of other factors, such as family environment and socioeconomic status, on these relationships. Despite the limitations, this study also has several strengths, including its representative sample of adolescents with depressive disorders who experienced home quarantine during the Covid-19 pandemic, the rigorous assessment process, and the adjustment for important confounding variables. The study’s findings shed light on the associations between NSSI and various factors, such as low self-esteem, sleep disturbance, and internet use, which can be useful for clinicians in diagnosing and preventing NSSI in patients. They can identify early-stage patients based on the results of this study and provide targeted interventions and treatments promptly. This may involve the use of Cognitive Behavioral Therapy (CBT), medication, and lifestyle adjustments to address the specific needs of the patients.

## 5 Conclusion

This study highlights that depressed adolescents engaging in NSSI often refrain from seeking help. Identifying specific risk factors is crucial as it allows for the development of preventive measures and targeted interventions. Clinical doctors can choose different treatment modalities, such as CBT and supervised pharmacotherapy (50), based on the individual circumstances of the patient. This emphasizes the importance of employing a nuanced, patient-centric approach in intervention strategies. These measures are not only relevant to addressing NSSI but are also vital for suicide prevention efforts. The findings of our research reveal that low self-esteem, internet use and sleep disturbance act as risk factors for NSSI behaviors in depressed adolescents.

Building upon these identified risk factors, a clinical risk assessment model was formulated during the study. Implementing this model enables effective identification of depressed adolescents prone to NSSI, allowing for timely and targeted interventions. Early interventions for those with these risk factors may contribute to a reduction in NSSI behaviors among this vulnerable group. Consequently, utilizing this clinical risk assessment model holds significant promise for enhancing early detection and prevention strategies and ultimately improving the prognosis of NSSI in adolescents with mood disorders.

## Data availability statement

The original contributions presented in the study are included in the article/supplementary material, further inquiries can be directed to the corresponding authors.

## Ethics statement

The studies involving humans were approved by Ethics Committee of Shandong Mental Health Centre. The studies were conducted in accordance with the local legislation and institutional requirements. Written informed consent for participation was not required from the participants or the participants’ legal guardians/next of kin in accordance with the national legislation and institutional requirements. Written informed consent was obtained from the individual(s) for the publication of any potentially identifiable images or data included in this article.

## Author contributions

ZL: Conceptualization, Data curation, Formal Analysis, Investigation, Methodology, Writing – original draft, Writing – review & editing. YW: Data curation, Formal Analysis, Writing – review & editing, Investigation. YY: Funding acquisition, Investigation, Project administration, Writing – review & editing. LK: Funding acquisition, Project administration, Supervision, Validation, Writing – review & editing.
