# Supplementary material for: Unveiling a novel clinical risk assessment model for identifying non-suicidal self-injury risks in depressed Chinese adolescents amidst the COVID-19 pandemic: insights from low self-esteem, internet use, and sleep disturbance
Source: Front Psychiatry. 2024 Jan 5;14:1259909. doi: 10.3389/fpsyt.2023.1259909 (PMC10797052; doi:10.3389/fpsyt.2023.1259909)
Supplement: Supplementary file 1 [file Table_1.DOCX]

*Supplementary Materials*

Association of emotion regulation with depressive symptoms in adolescent girls: The mediating and moderating roles of self-esteem and peer rejection

Table of contents

1. Additional Results 2

**Table S1.** Comparison of homogeneity of general information between test set and validation set.2

**Table S2.** Comparison of test set and validation group homogeneity in SAD, CDI, IAT, DS and sleep disorders.3

| **Variable** | | **Testing set**  **n=302(%)** | |  | **Validation set**  **n=164(%)** | |  | ***χ²*/*t*** |
| --- | --- | --- | --- | --- | --- | --- | --- | --- |
| Gender | |  |  |  |  |  |  | 6.86^*^ |
| Male | | 74(24.5) | |  | 59(36.0) | |  |  |
| Female | | 228(75.5) | |  | 105(64.0) | |  |  |
| Education | |  |  |  |  |  |  | 1.52 |
| Junior high school and below | | 144(47.7） | |  | 88(53.7） | |  |  |
| High school and above | | 158(52.3） | |  | 76(46.3） | |  |  |
| Family history of psychosis | |  |  |  |  |  |  | 1.95 |
| Without |  | 271(89.7) | |  | 140(85.4) | |  |  |
| With |  | 31(10.3) | |  | 24(14.6) | |  |  |
| Family location |  |  |  |  |  |  |  | 0.15 |
| Urban |  | 201(66.6) | |  | 112(68.3) | |  |  |
| Rural |  | 101(33.4) | |  | 52(31.7) | |  |  |
| Have siblings or not |  |  |  |  |  |  |  | 0.61 |
| YES |  | 197(65.2) | |  | 101(61.6) | |  |  |
| NO |  | 105(34.8) | |  | 63(38.4) | |  |  |
| Marital status of biological | |  |  |  |  |  |  | 0.53 |
| Normal |  | 265(87.7) | |  | 140(85.4) | |  |  |
| Divorced |  | 28(9.3) | |  | 18(11.0) | |  |  |
| Unilateral alive |  | 9(3) | |  | 6(3.7) | |  |  |
| Financial status |  |  |  |  |  |  |  | 0.98 |
| Well |  | 60(19.9) | |  | 39(23.8) | |  |  |
| Medium |  | 179(59.3) | |  | 92(56.1) | |  |  |
| Worse |  | 63(20.9) | |  | 33(20.1) | |  |  |
|  |  | Mean±SD | |  | Mean±SD | |  |  |
| Age | | 15.1±1.8 | |  | 15.1±1.8 | |  | -0.35 |

**Table S1** Comparison of homogeneity of general information between test set and validation set

NOTE: NSSI, patients with non-suicidal self-injury. **:*p*<0.001.*:*p*<0.05.

**Table S2** Comparison of test set and validation group homogeneity in SAD, CDI, IAT, DS and sleep disorders

| **The Scale** | **Testing set**  **n=302** | **Validation set**  **n=164** | ***t*** | ***p*** |
| --- | --- | --- | --- | --- |
| Social distress | 9.9±4.2 | 9.6±4.2 | 0.82 | >0.05 |
| Social avoidance | 9.5±4.1 | 9.3±4.1 | 0.48 | >0.05 |
| CDI 1 | 5.5±3.7 | 6.1±3.5 | -1.83 | >0.05 |
| CDI 2 | 2.3±1.7 | 2.4±1.7 | -0.47 | >0.05 |
| CDI 3 | 5.0±2.5 | 5.2±2.5 | -0.97 | >0.05 |
| CDI 4 | 4.8±2.1 | 4.9±1.9 | -0.56 | >0.05 |
| CDI 5 | 7.5±3.9 | 7.7±4.0 | -0.55 | >0.05 |
| IAT | 46.3±17.4 | 45.5±16.5 | 0.49 | >0.05 |
| Sleep disturbance | 9.6±6.5 | 9.5±6.7 | 0.29 | >0.05 |
| DS | 36.4±17.3 | 37.3±16.4 | -0.57 | >0.05 |

NOTE: CDI: Children’s Depression Inventory, IAT: The Internet Addiction Test, DS: Defeat Scale, CDI 1= CDI negative affect, CDI 2= CDI interpersonal problems, CDI 3= CDI low self-esteem, CDI 4= CDI low efficacy, CDI 5= CDI lack of pleasure
